# Piezoelectric Response of Multi-Walled Carbon Nanotubes

**DOI:** 10.3390/ma11040638

**Published:** 2018-04-21

**Authors:** Marina V. Il’ina, Oleg I. Il’in, Yuriy F. Blinov, Alexey A. Konshin, Boris G. Konoplev, Oleg A. Ageev

**Affiliations:** 1 Electronics and Electronic Equipment Engineering, Institute of Nanotechnologies, Southern Federal University, 347922 Taganrog, Russia; oiilin@sfedu.ru (O.I.I.); blinovyf@sfedu.ru (Y.F.B.); 2 Research and Education Center “Nanotechnologies”, Southern Federal University, 347922 Taganrog, Russia; konshin@sfedu.ru (A.A.K.); kbg@sfedu.ru (B.G.K.).

**Keywords:** nanoelectronics, nanopiezotronics, carbon nanotubes, piezoelectric effect, flexoelectric effect, strain, scanning probe microscopy

## Abstract

Recent studies in nanopiezotronics have indicated that strained graphene may exhibit abnormal flexoelectric and piezoelectric properties. Similar assumptions have been made with regard to the properties of carbon nanotubes (CNTs), however, this has not so far been confirmed. This paper presents the results of our experimental studies confirming the occurrence of a surface piezoelectric effect in multi-walled CNTs under a non-uniform strain. Using atomic force microscopy, we demonstrated the piezoelectric response of multi-walled CNTs under compression and bending. The current generated by deforming an individual CNT was shown to be −24 nA. The value of the surface potential at the top of the bundle of strained CNTs varied from 268 mV to −110 mV, depending on strain type and magnitude. We showed that the maximum values of the current and the surface potential can be achieved when longitudinal strain predominates in a CNT. However, increasing the bending strain of CNTs does not lead to a significant increase in current and surface potential, due to the mutual compensation of piezoelectric charges concentrated on the CNT side walls. The results of the study offer a number of opportunities and challenges for further fundamental research on the piezoelectric properties of carbon nanotubes as well as for the development of advanced CNT-based nanopiezotronic devices.

## 1. Introduction

Nanoscaling of electronics has led to the significant influences of the flexoelectric effect, which determines the relationship between polarization and strain gradient, and the surface piezoelectric effect, which is negligible in bulk materials due to the small “surface/volume” ratio on the electromechanical properties of materials [1,2,3]. Consequently, a new area of modern electronics has emerged, i.e., nanopiezotronics [4], which utilizes the coupling of flexoelectronic and piezoelectric properties of nanostructures to develop new tools and devices. The basic element of nanopiezotronics is a two-electrode structure, in which the internal electric field occurring in a nanostructure under strain is used as a voltage, controlling the charge carriers transport [5,6,7,8,9,10]. The fundamental principles of nanopiezotronics were developed less than ten years ago [11]; however, the search for materials for practical implementation of this area of research continues to the present.

This area of research considers not only nanostructures based on traditional piezoelectric materials, but also those based on materials that do not exhibit volumetric piezoelectric properties [5]. The reason for this is electronic polarization caused by the appearance of the flexoelectrical effect that can arise in nanostructures in the formation of their strain gradient, regardless of the lattice symmetry type [1,12]. This area is specifically focused on carbon nanostructures, which, being centrosymmetric materials, still exhibit anomalous flexoelectric and piezoelectric properties [3,13,14,15]. A well-established technology for obtaining carbon nanostructures, as well as high values of strength and elasticity, and allowing 25% strain, make them a promising material for nanopiezotronics. At this stage, it has been experimentally and theoretically confirmed that graphene is polarized under strain as a result of the appearance of flexoelectric and surface piezoelectric effects [3,13]. In this case, the mechanism for the appearance of the surface piezoelectric effect of graphene differs from the classical volume effect and, presumably, is related to the asymmetric redistribution of the electron density while graphene is under strain. This in turn leads to the formation of a region with a low carrier density and a significant increase in the screening length [3].

Recent theoretical studies have shown that a similar redistribution of electron density is also observed when a graphene sheet is twisted to form a carbon nanotube [14,16,17]. Each carbon atom of the nanotube forms its electric moment [14], while the total internal electric field of the CNT is zero because of its cylindrical symmetry [16]. However, breaking the cylindrical symmetry of CNTs can lead to significant polarization [16]. The above studies allowed us to conclude that a non-uniform strain of CNTs and, consequently, the violation of its cylindrical symmetry will lead to the appearance of flexoelectric and/or surface piezoelectric effects and the appearance of an internal electric field in the nanotubes [18,19]. Our study showed that the piezoelectric coefficient of the CNT is 0.107 ± 0.032 C/m^2^ [19], which is comparable to the piezoelectric coefficient of the basic piezoelectric nanomaterials [2]. These results became the basis of our research and development of memristive structures using CNTs under a non-uniform strain [18]. We found that memristive switching of a carbon nanotube is related to the formation and subsequent redistribution of a non-uniform elastic strain and piezoelectric charge in a nanotube under the influence of an external electric field [18,19,20,21,22]. In addition, the presence of flexoelectric and piezoelectric properties in CNTs could explain the phenomenon of hysteresis of the current-voltage characteristics in the study of the emission properties of vertically aligned carbon nanotubes (VA CNTs) and electric parameters of nanotube bundles, which still has not been unambiguously explained [22,23,24,25].

The purpose of this paper is to describe the experimental confirmation of the surface piezoelectric effect in multilayer carbon nanotubes, and to determine the influence patterns of the effect of the magnitude and type of CNT strain on the level of the generated current and surface potential.

## 2. Materials and Methods

### 2.1. Experimental Samples

**Figure 1:**
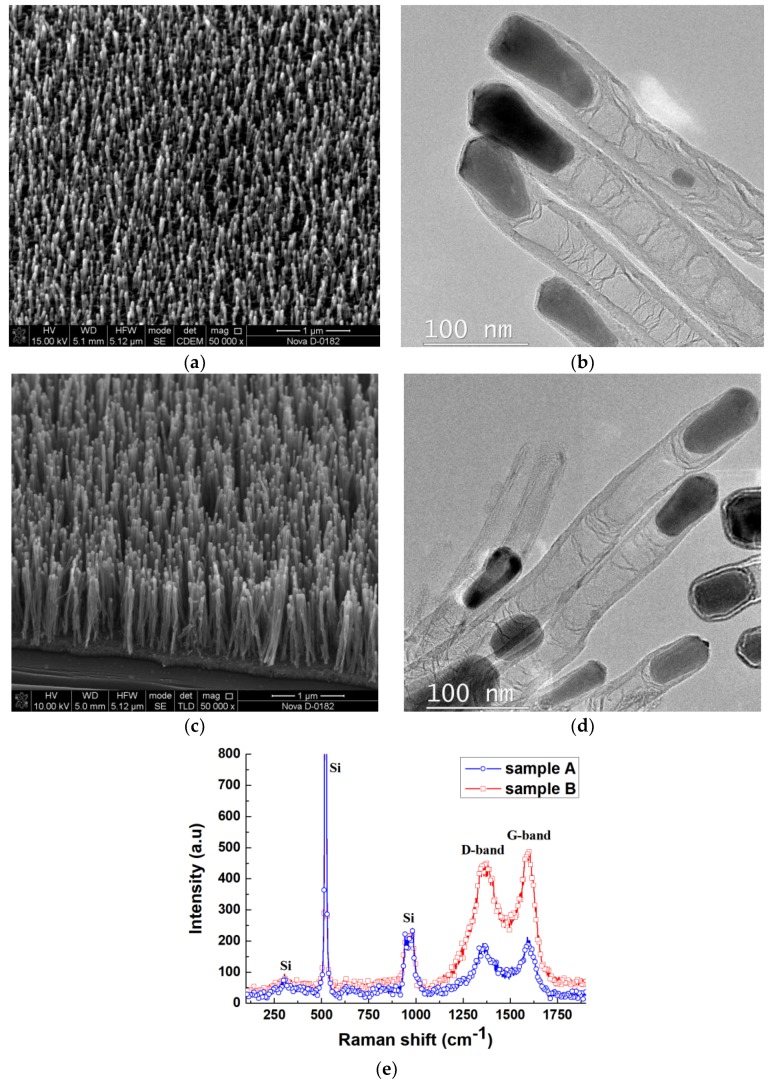
The experimental samples of vertically aligned carbon nanotube arrays: (**a,c**) SEM images of samples A and B, respectively; (**b,d**) TEM images of samples A and B, respectively; (**e**) Raman spectra of samples A and B.

We used two types of VA CNT arrays as experimental samples (Figure 1). A wafer of chemically purified silicon Si(100) was used as the substrate for the experimental samples, barrier (TiN), and catalyst (Ni) with thicknesses of 100 and 10 nm, respectively, which were formed by magnetron sputtering. Then the substrate was scribed into individual samples. VA CNTs were formed by the method of plasma-enhanced chemical vapor deposition (PECVD) using the nanotechnology multi-functional complex NANOFAB NTC-9 (NT-MDT, Moscow, Russia). The formation of Ni catalytic centers, from the continuous film of the catalytic material, was carried out by heating the samples to 660 °C within 20 min in an argon (40 sccm) and ammonia (15 sccm) atmosphere at a pressure of 4.5 Torr. Then the substrate was held in ammonia plasma (210 sccm) for 30 s and 2 min for samples A and B, respectively. Plasma was initiated using a high-voltage DC source. After that, acetylene (70 sccm), together with ammonia, were added into the chamber and VA CNTs were produced by a tip-growth mechanism for 15 min.

The geometrical parameters of the grown VA CNT arrays were measured using the scanning electron microscope (SEM) Nova NanoLab 600 (FEI, Eindhoven, The Netherlands) (Figure 1a,c). The structural analysis of the samples was conducted by transmission electron microscopy (TEM) using a Tecnai Osiris (FEI, Eindhoven, The Netherlands) (Figure 1b,d) and a Raman spectrometer Renishaw InVia Reflex (Renishaw plc, Wotton-under-Edge, UK) with laser excitation wavelengths of 514.5 nm (Figure 1e).

### 2.2. Measurement Methods

**Figure 2:**
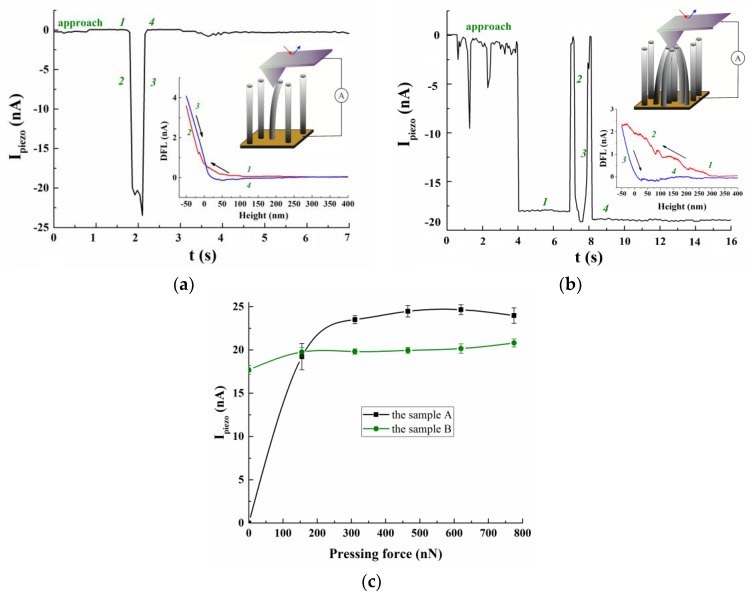
The results of the piezoelectric response study: (**a**) an individual carbon nanotube of sample A; (**b**) a bundle of strained CNTs of sample B. The inserts show schematic images of the measurement process and the results of AFM force spectroscopy: 1—force is not applied to the AFM probe, 2—gradual force is applied to the AFM probe, 3—gradual force decay back to zero, 4—the AFM probe returns to initial state 1; (**c**) the relationship between the detected current and the applied force of the AFM probe.

Experiments were held by atomic force microscopy (AFM) using the Ntegra probe nanolaboratory (NT-MDT, Moscow, Russia). Measurements of the piezoelectric response of CNTs included detection of the current generated in the “lower electrode/CNT/AFM probe” system during the elastic strain of a nanotube or a bundle of nanotubes under mechanical pressure from the AFM probe on their tops. The barrier layer of TiN served as the lower electrode. A commercial cantilever, with a platinum coating of NSG11/Pt, was used as the AFM probe. CNT bundles were formed by mechanical swaying of the nanotubes and combining them via van der Waals forces during scanning in the AFM semi-contact mode. A subsequent elastic strain of CNTs was performed in the AFM force spectroscopy mode. Schematic representations of the process of force spectroscopy of CNTs for samples A and B are presented in the insets of Figure 2 a,b, respectively.

**Figure 3:**
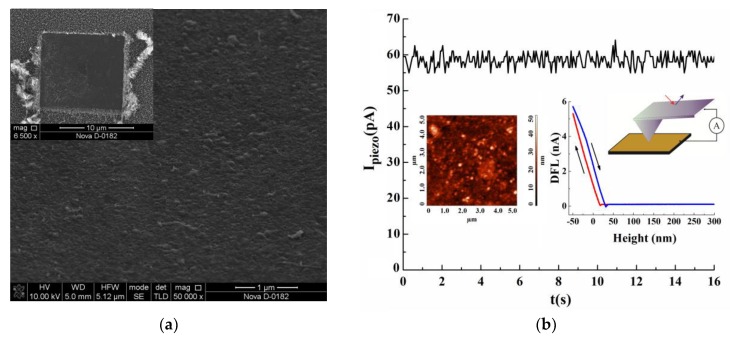
The results of the piezoelectric response of the mechanically purified substrate: (**a**) a SEM image of the purified area. The inset is a SEM image of the 20 × 20 μm^2^ area of the array and was scanned in the AFM force lithography mode; (**b**) the current-time correlation of the substrate straining in the AFM force spectroscopy mode. The inset shows an AFM image of the research area, schematic image of the measurement process, and the results of AFM force spectroscopy.

To exclude the influence of the AFM measurement system, the substrate and lower electrode materials on the measurement results—with similar measurements for the piezoelectric response—were carried out on a substrate that was mechanically purified from nanotubes. For this purpose, the 20 × 20 μm^2^ area of the array was scanned in the AFM force lithography mode, and as a result, the carbon nanotubes broke away from the substrate and moved outside the scanned area (the insert in Figure 3a). Within this region, a 10 × 10 μm^2^ area was then re-scanned to completely remove the torn off nanotubes from the substrate surface. The SEM image of the 10 × 10 μm^2^ area, cleared of CNTs, is shown in Figure 3a.

**Figure 4:**
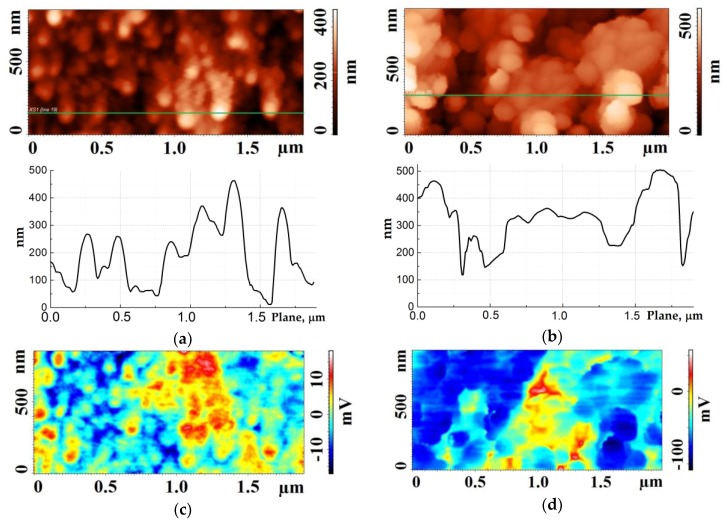
AFM scanning of the surface potential of elastically strained CNTs: (**a**,**b**) topology of the surface (top) and cross section along the line (bottom) of sample A and sample B, respectively; (**c**,**d**) potential distribution of sample A and sample B, respectively.

Measurements of the surface potential were performed in the Kelvin probe mode of the AFM. On the first pass, preliminary scanning of the surface of the experimental samples in the semi-contact AFM mode, with AFM probe oscillation amplitudes of 35 to 195 nm, was performed. During the scanning process, carbon nanotubes combined into bundles of different diameters via van der Waals forces (Figure 4) [22]. Measurement of the surface potential of elastically strained CNTs forming bundles was performed on the second pass at an 11 nm distance between the surface of the CNT array and an AFM probe. No external electric field between the probe and the CNT array was applied.

## 3. Results and Discussion

### 3.1. Characterization of CNTs

The analysis of the SEM images of the grown VA CNT arrays showed that the diameter, length, and density of CNTs in sample A were almost 37 ± 3 nm, 370 ± 40 nm, and 47 μm^−2^, respectively (Figure 1a). In sample B it was shown that the diameter, length, and density of CNTs were almost 43 ± 7 nm, 1.4 ± 0.2 μm, and 128 μm^−2^, respectively (Figure 1c). Analysis of TEM images showed that the experimental samples were multi-walled carbon nanotubes with bamboo-like defects (Figure 1b,d). The multi-walled structure of CNTs suggests that they have a metallic type of conductivity, as at least one sheet has a metallic chirality. The Raman spectra of samples A and B (Figure 1e) showed the presence of D- and G-modes, which is typical for multi-walled aligned carbon nanotubes [26]. The high intensity of the D-mode can be associated with defective nanotube structures such as defects of a nanotube’s cap, the presence of broken bonds, and amorphous carbon [26].

### 3.2. Piezoelectric Response of CNTs

The results of the study on the piezoelectric response of individual CNTs in sample A showed that when the AFM probe is applied to the top of the nanotube, the current in the “lower electrode/CNT/AFM probe” system does not flow, since in the initial state, the CNT does not undergo strain (section 1 in Figure 2a). With a gradual force application of 0 to 155 nN, a current of 0 to −22 nA, respectively, arises in the system (section 2 in Figure 2a). When the force decreases from 155 to 0 nN at the initial instant of time (*t* = 2.1 s), there is a small surge of current up to −24 nA, followed by its gradual decay back to zero (section 3 in Figure 2a). The surge of current at *t* = 2.1 s is probably caused by changing the type of strain of CNTs from bending-strain to stress-strain under the action of adhesion forces holding the CNT on top of the AFM probe during retraction at the initial instant of time [27]. Thus, when bending the CNT, stretching strain with a positive charge arises on one side of the top of the nanotube, and compressive strain with a negative charge occurs on the other, reducing the total piezoelectric charge [4,28]. With the subsequent stretching of the nanotube, a positive piezoelectric charge begins to predominate at its top, which results in the detection of a small increase in current.

Measurements of the piezoelectric responses of the CNT bundles in sample B showed that when the AFM probe is applied to the bundle top in the “lower electrode/bundle CNT/AFM probe” system, a current of −17 nA flows, as in the initial state, the nanotubes forming the bundle undergo elastic bending strain (section 1 in Figure 2b). With the subsequent application of 0 to 155 nN force to the probe, a current of 0 to −19 nA arises in the system (section 2 in Figure 2b). At the same time, the maximum value of the current achieved with a force of 155 nA was smaller for the CNT bundle than for an individual nanotube. This may be because the nanotubes forming the bundle experienced greater bending strain than individual CNTs, which resulted in a decrease in the total piezoelectric charge at their tops. With a subsequent decrease in the applied force and removal of the AFM probe from the bundle top, the current value dropped to zero (section 3 in Figure 2b). After the force spectroscopy, the AFM probe was once again brought close to the top, and again a current of about −18 nA was detected in the system (section 4 in Figure 2b).

Subsequent increases in the AFM probe force, applied to the top of an individual CNT from 155 to 800 nN, showed that the value of the detected current gradually saturates (Figure 2c). This dependence may be explained by the fact that when a weak force (up to 200 nN) is applied, the nanotube predominantly experiences compressive strain and only a negative piezoelectric charge is formed at its top. Increasing the force further, the bending strain begins to predominate, which leads to the formation of simultaneously positive and negative charges on the top. As a result, when the force is increased, the total piezoelectric charge varies insignificantly, and the current-force dependence saturates. A similar outcome was observed for CNT bundles, where the current shows almost no growth as the force increases, which is due to the predominance of bending strain (Figure 2c).

Measurements of the piezoelectric response on the substrate, with nanotubes removed for each experimental sample, confirmed the absence of a piezoelectric response in the “lower electrode/substrate/AFM probe” system. Thus, it was found that when the AFM probe was pressed against the purified substrate in the “lower electrode/substrate/AFM probe” system, an insignificant current flowed, the value of which did not correlate with the force of pressing, and was about 60 pA (Figure 3b). The detected current can be attributed to the measuring system tolerance of the probe nanolaboratory Ntegra.

Thus, it can be concluded that the current arising in the “lower electrode/CNT/AFM probe” system during the CNTs’ strain is not related to the formation properties of the substrate, or the lower and upper electrodes, and can only be explained by the piezoelectric properties of the carbon nanotubes.

Such anomalous piezoelectric properties of carbon nanotubes are related to the non-uniform strain occurring in the process of AFM force spectroscopy. The non-uniform strain of CNTs leads to an asymmetric redistribution of the electron density along the nanotube axis and the formation of a nonzero total electric moment in the nanotube, similar to the effect previously observed in strained graphene [3,14]. In addition, other reasons for the violation of the central symmetry of CNTs include the presence of the nickel catalytic center on its apex and bamboo-like structural defects that can increase the value of the total electric moment of the strained CNT. The multi-walled carbon nanotube can also enhance its piezoelectric properties by summing the electrical moments of each layer. We assume that there should be an optimal number of CNT layers, at which the maximum piezoelectric response will be observed. However, this assumption requires further research.

In addition, it is necessary to take into account that the CNTs’ strain not only leads to the appearance of the piezoelectric effect but also to the piezoresistive effect associated with the change in the band gap and the conductivity of the nanotube [29,30,31].

### 3.3. Surface Potential of Elastically Strained CNTs

Studies of the surface potential of CNT arrays of experimental samples A and B also confirmed the manifestation of piezoelectric properties of CNTs under strain. Thus, after scanning sample A in a semi-contact AFM mode, neighboring individual CNTs formed bundles of 2 to 3 nanotubes with a diameter varying from 78 to 110 nm (Figure 4a). A positive potential of up to 18 mV was formed on the tops of these CNT bundles, while at the bottom we registered a negative potential—up to −17 mV (Figure 4c). It can be assumed that the nanotubes, which were combined into bundles, experienced a predominantly stretching strain, which led to the formation of a positive potential. Thus, we can conclude that a bundle of 2–3 nanotubes is an electric dipole with a positive charge concentrated at the top, and a negative one at the base.

Scanning sample B, which had a much higher density of nanotubes, led to the formation of CNT bundles with diameters from 87 to 290 nm (Figure 4b). At their tops, a potential was formed from 268 to −110 mV (Figure 4d). The positive potential was observed at the tops of the CNT bundles up to 130 nm in diameter, which also corresponded to a bundle of 2–3 nanotubes. The negative potential was formed at the tops of the large diameter bundles, which may be due to the prevalence of bending strain in nanotubes when the diameter of the bundle is greater than 130 nm [28,32].

In addition, a further increase in the average diameter of the CNT bundles of sample B, achieved by increasing the amplitude of the AFM probe oscillation during the scanning of the CNT array in a semi-contact mode, led to a decrease in the value of the surface potential at their tops. An increase in the amplitude of the AFM probe oscillation from 35 to 195 nm, allowed the attainment of CNT bundles with average diameters from 110 to 460 nm. The resulting average value of the surface potential at the tops of the CNT bundles of about 150 nm in diameter was −115 ± 7 mV. At the tops of the CNT bundles of about 200 nm in diameter, the potential decreased to −84 ± 4 mV, while on bundles with a diameter of about 460 nm, it was −64 ± 2 mV. Thus, a larger diameter of the CNT bundles leads to a decrease in the average value of the surface potential, which is associated with an increase in CNT bending strain and a decrease in the magnitude of the total piezoelectric charge formed at the top of the bundle.

Thus, we showed that the value of the surface potential depends on the diameter of the CNT bundle and, accordingly, on the type and magnitude of strain of its nanotubes, which agrees with the results of our previous studies [18,19]. In order to obtain the maximum value of the surface potential, it is preferable to use an array of vertically aligned carbon nanotubes, while application of external mechanical force will primarily lead to the formation of a longitudinal strain, which should be taken into account when developing highly efficient CNT-based nanogenerators.

## 4. Conclusions

This article comprises the study of two arrays of vertically aligned multi-walled nanotubes with different geometric parameters. The experiments showed that a piezoelectric current and surface potential appear in the CNTs of each sample, with their values depending on the magnitude and type of strain. Analysis of the obtained results allows us to draw the following conclusions:

(1) It is now empirically supported that there is a surface piezoelectric effect in multi-walled carbon nanotubes (earlier the effect only had a predictive character [14,18]).
(2) It is shown that the maximum values of current and potential are reached when the CNT undergoes a longitudinal compressive or stretching strain. In this context, the CNT can be considered as a single electric dipole with charges concentrated at its ends.
(3) With increased bending of CNTs, the values of a piezoelectric current and potential vary insignificantly, which is related to an insignificant increase in the total piezoelectric charge concentrated at their tops, due to a partially mutual compensation of charges concentrated on the CNT side walls.

The results of this study offer a number of opportunities for further fundamental and applied research on the piezoelectric properties of carbon nanotubes and the development of advanced CNT-based nanopiezotronic devices. In particular, the results can be used to create nonvolatile memory elements based on carbon nanotubes, which exceed the currently existing elements in speed, scalability, and energy efficiency. In addition, the ability of carbon nanotubes to transform external mechanical influences, including minor mechanical vibrations of the ambient environment into electrical current, can be used as a basis for creating highly efficient nanogenerators and elements of energy conversion and storage [33,34,35,36].

However, there also challenges to be addressed, in particular, related to the influence of chirality, the number of layers, and the defects of carbon nanotubes on their piezoelectric properties. Our further research will be focused on solving these issues and developing promising two-electrode nanopiezotronic structures based on the piezoelectric properties of CNTs.

The results were obtained using the equipment of the Research and Education Center and the Center for Collective Use of “Nanotechnologies” at the Southern Federal University.
