# Erratum: Il’ina, M.V.; et. al. Piezoelectric Response of Multi-Walled Carbon Nanotubes. *Materials* 2018, *11*, 638

**DOI:** 10.3390/ma12010165

**Published:** 2019-01-07

**Authors:** Materials Editorial Office

**Affiliations:** MDPI, St. Alban-Anlage 66, 4052 Basel, Switzerland; Tel.: +41-61-683-7734

The *Materials* Editorial Office would like to report errors in the published paper [1]. The details are as follows: 

Affiliation 1 of the authors should be changed to “Institute of Nanotechnologies, Electronics and Electronic Equipment Engineering, 347922 Taganrog, Russia”.

We apologize for any inconvenience caused to the readers by this change. The change does not affect the scientific results. The manuscript will be updated and the original will remain available on the article webpage.
